# Production of IL-8, VEGF and Elastase by Circulating and Intraplaque Neutrophils in Patients with Carotid Atherosclerosis

**DOI:** 10.1371/journal.pone.0124565

**Published:** 2015-04-20

**Authors:** Franca Marino, Matteo Tozzi, Laura Schembri, Stefania Ferraro, Antonino Tarallo, Angela Scanzano, Massimiliano Legnaro, Patrizio Castelli, Marco Cosentino

**Affiliations:** 1 Center of Research in Medical Pharmacology, University of Insubria, Varese, Italy; 2 Department of Surgical and Morphological Sciences, University of Insubria, Varese, Italy; University of Udine, ITALY

## Abstract

**Objectives:**

Polymorphonuclear neutrophils (PMN) in atherosclerotic plaques have been identified only recently, and their contribution to plaque development is not yet fully understood. In this study, production of elastase, interleukin (IL)-8 and vascular endothelial growth factor (VEGF) by PMN was investigated in subjects with carotid stenosis undergoing carotid endarterectomy (CEA).

**Methods:**

The study enrolled 50 patients (Pts) and 10 healthy subjects (HS). Circulating PMN (cPMN) isolated from venous blood (in both Pts and HS) and from plaques (pPMN, in Pts) were cultured, alone or with 0.1 μM fMLP. Elastase, IL-8 and VEGF mRNA were analyzed by real-time PCR. In CEA specimens, PMN were localized by immunohistochemistry.

**Results:**

In both Pts cPMN and pPMN, IL-8 mRNA was higher at rest but lower after fMLP (P<0.01 vs HS), and VEGF mRNA was higher both at rest and after fMLP (P<0.01 vs HS), while elastase mRNA was not significantly different. On the contrary, protein production was always higher in cPMN of HS with respect to values measured in cells of Pts. In CEA specimens, CD66b+ cells localized to areas with massive plaque formation close to neovessels. Pts with soft and mix plaques, as defined by computed tomography, did not differ in cPMN or pPMN IL-8, VEGF or elastase mRNA, or in intraplaque CD66b+ cell density. However, Pts with soft plaques had higher white blood cell count due to increased PMN.

**Conclusions:**

In Pts with carotid plaques, both circulating and intraplaque PMN produce IL-8, VEGF and elastase, which are crucial for plaque development and progression. These findings suggest mechanistic explanations to the reported correlation between PMN count and cardiovascular mortality in carotid ATH.

## Introduction

Atherosclerosis (ATH) is associated with the accumulation of cholesterol deposits in subendothelial macrophage-derived foam cells and by subsequent adherence and entry of leukocytes into the arterial wall, migration of smooth muscle cells into the intima, activation and aggregation of platelets, endothelial dysfunction, and the production of inflammatory cytokines [1–3]. Among immune cells infiltrating atherosclerotic lesions, polymorphonuclear neutrophil leukocytes (PMN) have been only recently identified as key contributors to the pathogenesis and progression of ATH [4–6], despite their involvement in the chronic, low-grade inflammation occurring early in subjects at high risk to develop cardiovascular disease (CVD) was extensively characterized [7–10].

PMN infiltration occurs in chronically inflamed arteries, and animal models shows that circulating PMN are recruited into atherosclerotic lesions, while depletion of PMN reduces plaque formation [11]. PMN products such as the human neutrophil peptide have been proposed as biological markers and potential therapeutic targets in cardiovascular diseases [12] an increased number of circulating neutrophils is a well-known risk indicator of future cardiovascular outcomes [5,13]. Atherectomy specimens with plaque erosion or rupture show clear PMN infiltration and patients with unstable angina pectoris have increased PMN within pathological lesions with respect to patients with stable disease [14].

Several possible mechanisms of PMN-driven atherogenesis and plaque destabilization have been proposed [15,16] such as the production of mediators which contribute to ATH. PMN are the exclusive producers of the enzyme elastase [17], which contribute to matrix degradation and weakening of the vessel wall associated with complications like aneurysm formation and plaque rupture [18].

PMN also produce high amounts of interleukin (IL)-8, a potent proinflammatory CXC-chemokine that promote PMN chemotaxis and degranulation [19]. High plasma levels of IL-8 are associated with an increased risk to develop CVD [20], and we have shown that circulating PMN from subjects at high cardiovascular risk exhibit increased production of IL-8 [7,9] while PMN from patients with peripheral arterial disease undergoing femoral endarterectomy produce only low amounts of IL-8 [21]. Recently IL-8 has been characterized in cancer as a key proangiogenic factor [22] and extensive evidence supports the role of PMN in tumor angiogenesis through the production of IL-8 and of other proangiogenic factors such as vascular endothelial growth factor (VEGF) [23,24]. Despite the importance of angiogenesis for atherogenesis and plaque development and destabilization [25,26], only fragmentary evidence so far exist about the production of IL-8 and VEGF by PMN in ATH.

In the present study, production of IL-8, VEGF and elastase, by PMN was investigated in patients with carotid stenosis undergoing carotid endarterectomy (CEA). PMN were isolated from both peripheral venous blood and surgically removed plaques, and their functional profiles were characterized. We aimed to compare the functional profile of PMN from patients (Pts) with ATH with cells from healthy subjects (HS) and to assess, in Pts, any possible differences between intraplaque PMN (pPMN) and cPMN. In addition, any possible correlations between PMN and plaque characteristics were evaluated. Finally, in a subgroup of surgical specimens, PMN were directly localized by immunohistochemistry and their preferential intraplaque localization was characterized.

## Materials and Methods

### Subjects

Consecutive Pts undergoing CEA were enrolled at the Unit of Vascular Surgery (Hospital di Circolo and Fondazione Macchi, Varese, Italy). Inclusion criteria were: carotid plaque with soft/fatty or mixed characteristics according to instrumental evaluation (see below) and eligible to CEA due to carotid stenosis ≥70% (even in the absence of cerebrovascular symptoms) or ≥50% (only in the presence of cerebrovascular symptoms), according to the American Heart Association guidelines [27,28]. Exclusion criteria were: carotid plaque with calcified characteristics, age >80 year old, recent infections (<1 month), history of autoimmune disease, cancer and/or chronic inflammatory disease, current or recent (<3 months) corticosteroid therapy, chronic alcoholism.

HS were enrolled among a population evaluated for a general clinical check-up and were included only if they fulfilled criteria for “low cardiovascular risk” classification according to the National Cholesterol Education Program—Adult Treatment Panel III (ATPIII) guidelines [29].

The study conforms to the principles outlined in the Declaration of Helsinki for use of human tissues or subjects and all the subjects gave verbal informed consent before enrolment. The study was performed on samples and data requested for the clinical management of patients, and no additional samples were obtained or additional procedures were performed. The study was approved by the ethics committee of the Hospital "di Circolo and Fondazione Macchi", Varese, Italy. The ethics committees of our Hospital (Ospedale di Circolo, Fondazione Macchi, Varese, Italy) approved this consent procedure.

Pts were evaluated by computed-tomography (CT), that represents an accurate modality to assess the presence of plaques and the severity of stenosis [30], allowing identification of calcification and/or thickening of the vessel wall and the length of the lesion (distance between the first (most proximal) and the last (most distal) image in which the lesion is present). Plaques were classified into 3 categories: fatty/soft (<60 Hounsfield units (HU)), mixed (60≤HU <129) and calcified (≥130 HU) [31]. Presence or absence of cerebrovascular symptoms was also considered [32] and subjects were grouped into symptomatic (presence of cerebrovascular symptoms) and asymptomatic (absence of cerebrovascular symptoms) and for all symptomatic patients the event of surgery was scheduled considering at least 6 months of attendance.

CEA was performed according to the European Vascular Surgery guidelines [33]. At the end of surgery, tissue samples were removed, signed onto the cranial extremity, dissected longitudinally and placed either in cold phosphate buffered saline (PBS) for subsequent isolation of PMN or in 4% paraformaldehyde for immune-cytochemical assays.

### Immunohistochemistry

CEA samples were divided into three parts: (i) areas with massive plaque formation (apart from the plaque core, usually completely calcified and not evaluable), (ii) areas adjacent to the plaque corresponding to (iia) internal carotid artery and to (iib) common carotid artery, where the plaque was almost or completely absent. The segments were designated for both histology and immunohistochemistry and then embedded in paraffin. Some sections were stained with hematoxylin/eosin and analyzed using an optical microscope. Other sections were used for immunohistochemistry: sections were deparaffinized in xylene and grades of alcohol, then rehydrated in water. Antigen retrieval was performed by heating in a pressure cooker with EDTA for 15min. After a washing in TRIS buffered saline/Triton X-100 solution, sections were incubated in a blocking solution (PBS, bovine serum albumin [BSA], NaN_3_) for 10 min and then incubated overnight at 4°C with mouse anti-human CD66b antibody (1:50). Dako REAL Detection System/Alkaline Phosphatase/RED was used as revelation system. Negative control was obtained omitting primary antibody. All the sections were analyzed by means of light microscopy and evaluated by a semiquantitative method.

### Isolation of circulating PMN

cPMN were isolated from venous blood samples of Pts and HS obtained by use of heparinized vacuum tubes after a fasting night, between 8:00 and 9:00 AM. Blood samples from Pts were obtained the day of surgery, before administration of pre-operatory drugs. Cell were isolated as previously described^3^. Purity of the preparations, assessed by flow cytometry, was usually 98–99%, while viability was 95–98% and assessed by the trypan blue exclusion test. PMN were finally resuspended in RPMI at the concentration of 1x10^7^/ml for cell culture. Viability after 24 h culture was 96.1±3.9% (resting conditions) and 97.0±3.7% (stimulated).

### Isolation of PMN from carotid plaques

For the isolation of PMN from plaques (pPMN), the surgical samples were carefully washed in PBS (5 min, room temperature), fragmented and mechanically disaggregated. Finally the homogenate was washed in 10 ml RPMI at room temperature, centrifuged (10 min, 300 g) and resuspended in 10 ml RPMI. Samples were filtered through a 70 μm and then through a 35 μm cell strainer. Finally, the homogenate was centrifuged and pPMNs were isolated by immunomagnetic sorting using the CD15+ Positive Isolation kit (Miltenyi), according the manufacturer’s instructions; cells were clearly identifiable by optical microscopy both in cell homogenate before sorting and in purified samples. Purity of the preparations, assessed by optical microscopy, was usually 96.3±4.0%, while viability was 98.9±1.3% and assessed by the trypan blue exclusion test. Retrieved pPMN were resuspended in RPMI (1x10^7^/ml) for culture; cell viability after 24 h culture was 92.2±3.5%.

### PMN culture

Both cPMN and pPMN were cultured at the concentration of 1x10^7^/ml for 24 h under standard conditions in RPMI alone or in the presence of 0.1 μM f-Met-Leu-Phe (fMLP, Sigma-Aldrich), a chemotactic peptide acting on membrane receptors that result in PMN activation [34]. We selected fMLP for PMN stimulation according our previous paper in high risk subjects [7,9] and in PAD Pts [21] in which we have shown that PMN response to fMLP was quite different with respect to HS. At the end of the culture, cells and supernatants were harvested and stored at -80°C until assay.

### Real-time PCR analysis

Total RNA was extracted from 1x10^6^ cells by Perfect RNA Eukaryotic Mini kit (Eppendorf) and the amount of extracted RNA was estimated by spectrophotometry. Total RNA was reverse transcribed using the high-capacity cDNA Archive Kit, according to the manufacturer’s instructions and Real-time PCR was performed as previously described [21], by use of the primers shown in Table 1. Threshold cycle values for the genes of interest (Ct1) were calculated, normalized to the Ct for 18S RNA (housekeeping gene) (Ct2) and finally expressed as 2^-ΔCt^, where ΔCt = Ct2—Ct1.

**Table 1:** Real-Time PCR gene expression.

Gene Symbol	UniGene ID	Interrogated Sequence	Translate Protein	Exon Boundary	Assay Location	Amplicon Lenght	Annealing temperature (°C)	Efficiency (%)
		*RefSeq/GenBank mRNA*		*RefSeq/GenBank mRNA*	*RefSeq/GenBank mRNA*			
**IL-8**	Hs. 00174103_m1	NM_000584.3	NP_000575.1	1–2	222	101	60	100.02
**VEGF**	Hs. 00900055_m1	NM_001025366.2	NP_001020537.2	3–4	1352	59	60	99.99
**elastase**	Hs. 00357734_m1	NM_001972.2	NP_001963.1	3–4	402	66	60	100.06

### ELISA assays

IL-8, VEGF and elastase levels in supernatants of cultured PMN were quantified using a sandwich-type enzyme-linked immunoadsorbent assay (QuantikineELISA, R&D System). The limits of detection were 1 pg/ml for VEGF and IL-8 and 1 ng/ml for elastase.

### Statistical analysis

Data are presented as means±standard deviation (SD) or as median and interquartile range, as appropriate. Comparisons between groups were performed using the Mann Whitney test, while comparisons between dependent measures were performed using the Wilcoxon test. Analysis of the correlation between functional responses of PMN and clinical variables was performed by linear regression analysis (for continuous variables) or by the Mann Whitney test (for categorical variables), and to account for the number of comparisons statistical significance for correlations was set at P<0.001. Statistical analysis was performed using GraphPad Prism version 5.00 for Windows (GraphPad Software, San Diego, CA, USA, www.graphpad.com).

## Results

### Subjects

We enrolled 22 Pts with soft plaques and 28 with mix plaques (50 Pts: F/M: 11/39). In comparison with Pts with soft plaques, Pts with mix plaques were older (on average, +6.5 years), with less impaired fasting glucose (10.7% vs 45.4%) and with more pneumopathies (35.7% vs 9.1%). There were no differences however regarding gender, smoking habits, alcohol use, family history of CVD, diabetes, hypertension and other CVD (Table 2), as well as regarding drug treatments (Table 3).

**Table 2:** Clinical characteristics of Pts at enrollment.

	Total	Soft	Mix	P
n	50	22	28	
F/M	11/39	4/18	7/21	0.734
Age (years)	71.4±1.9	68.3±1.8	73.9±1.2	0.009
Smokers	36/50	17/22	19/28	0.537
Alcohol use^1^	3/50	3/22	0/28	0.079
Family history of CVD	35/50	18/22	17/28	0.155
Impaired fasting glucose^2^	35/50	10/22	3/28	0.009
Diabetes	14/50	6/22	8/28	1.000
Hypertension^3^	46/50	21/22	25/28	0.621
Other CVD	27/50	10/22	17/28	0.393
Pneumopathy	12/50	2/22	10/28	0.018

P indicates statistical significance of the differences between soft and mix plaques.

1 = >4 cups of wine/die or >2 cups of spirits/die.

2 = fasting blood glucose >110 mg/dl.

3 = according to the guidelines of the National Committee on Prevention, Detection, Evaluation, and Treatment of High Blood Pressure, National High Blood Pressure Education Program Coordinating Committee [29].

**Table 3:** Drug treatments of Pts at enrollment.

	Total	Soft	Mix	P
Antiplatelet drugs	42/50	18/22	24/28	0.718
Statins	33/50	14/22	19/28	0.773
β-blockers	16/50	7/22	9/28	1.000
ACE inhibitors/AT1R-antagonists	26/50	11/22	15/28	1.000
Ca^++^-channel blockers	16/50	7/22	9/28	1.000
Diuretics	9/50	3/22	6/28	0.713
Hypoglycemic drugs	5/50	3/22	2/28	0.643
Other drugs	27/50	13/22	14/28	0.577

P indicates statistical significance on the differences between soft and mix plaques.

Pts with soft plaques had higher systolic and diastolic blood pressure values, and white blood cells, possibly due to increased PMN. There were no differences regarding other clinical data (Table 4).

**Table 4:** Clinical data of Pts at enrollment.

	Total	Soft	Mix	P
Blood pressure (mm Hg)				
*- systolic*	130.0 (120.0–140.0)	140.0 (127.5–161.8)	130.0 (120.0–130.0)	0.004
*- diastolic*	80.0 (70.0–80.0)	80.0 (70.0–81.2)	70.0 (60.0–80.0)	0.016
BMI				
*- F*	25.0 (21.5–29.4)	25.03 (21.4–31.2)	22.9 (21.5–29.4)	0.788
*- M*	26.1 (24.9–29.2)	22.9 (21.5–29.3)	27.5 (25.6–30.1)	0.014
Cholesterol (mg/dl)				
*- total*	165.5 (144.8–199.5)	168.0 (141.0–220.0)	163.0 (146.0–189.0)	0.479
*- HDL*	43.0 (42.0–50.0)	44.0 (32.5–50.0)	43.0 (42.0–50.5)	0.837
*- LDL*	92.8 (72.0–118.4)	105.1 (79.0–132.9)	92.8 (64.8–106.3)	0.315
Triglycerides (mg/dl)	137.5 (97.5–181.5)	155.0 (109.0–199.0)	124.0 (88.5–170.0)	0.122
Hs-CRP (mg/dl)	4.6 (1.3–7.2)	6.0 (1.3–10.0)	3.9 (1.3–5.5)	0.311
Creatine kinase (UI/l)	98.0 (72.5–140.5)	90.0 (58.7–151.0)	108.0 (73.0–138.5)	0.749
Glycemia (mg/dl)	99.0 (89.0–119.0)	109.0 (85.0–123.0)	97.5 (90.5–105.0)	0.309
Red blood cells (10^6^/mm^3^)	4.6 (4.4–5.1)	4.8 (4.4–5.2)	4.6 (4.2–4.7)	0.110
White blood cells (10^3^/mm^3^)	6.9 (5.9–8.5)	7.4 (6.6–9.2)	6.3 (5.3–7.5)	0.002
PMN				
- n (10^3^/mm^3^)	4.0 (3.1–4.7)	4.2 (3.5–5.0)	3.7 (3.0–4.5)	0.740
- %	57.7 (50.6–62.4)	57.2 (52.9–63.5)	57.8 (50.1–62.1)	0.196
Lymphocytes				
- n (10^3^/mm^3^)	2.2 (1.7–2.5)	2.3 (1.7–2.7)	2.2 (1.7–2.3)	0.767
- %	29.7 (25.6–35.6)	28.7 (25.6–37.4)	30.2 (25.5–35.5)	0.268

Data are expressed as median (25^th^-75^th^ percentile); P indicates statistical significance on the differences between soft and mix plaques.

Stenosis was: 21 left, 28 right and 1 both sides (soft: 11 left and 10 right; mix: 10 left and 18 right; P = 0.262). Plaques were symptomatic in 19 cases (10 soft; 9 mix; P = 0.389) (Table 5). Enrolled HS were 10 (F/M: 3/7; P = 0.685 vs Pts) and were aged 66.4±3.9 years (P = 0.100 vs Pts).

**Table 5:** Characteristics of the plaques.

	Total	Soft	Mix	P
stenosis (left/right)				
*- total*	21/28*	11/10*	10/18	0.262
*- F*	4/6*	2/1*	2/5	0.500
*- M*	17/22	9/9	8/13	0.528
Symptomatic/asymptomatic				
*- total*	19/31	10/12	9/19	0.389
*- F*	7/4	4/0	3/4	0.194
*- M*	12/27	6/16	6/15	1.000

P indicates statistical significance on the differences between soft and mixed plaques.

* = 1 F with bilateral stenosis.

### Immunohistochemical identification of intraplaque PMN

**Fig 1:**
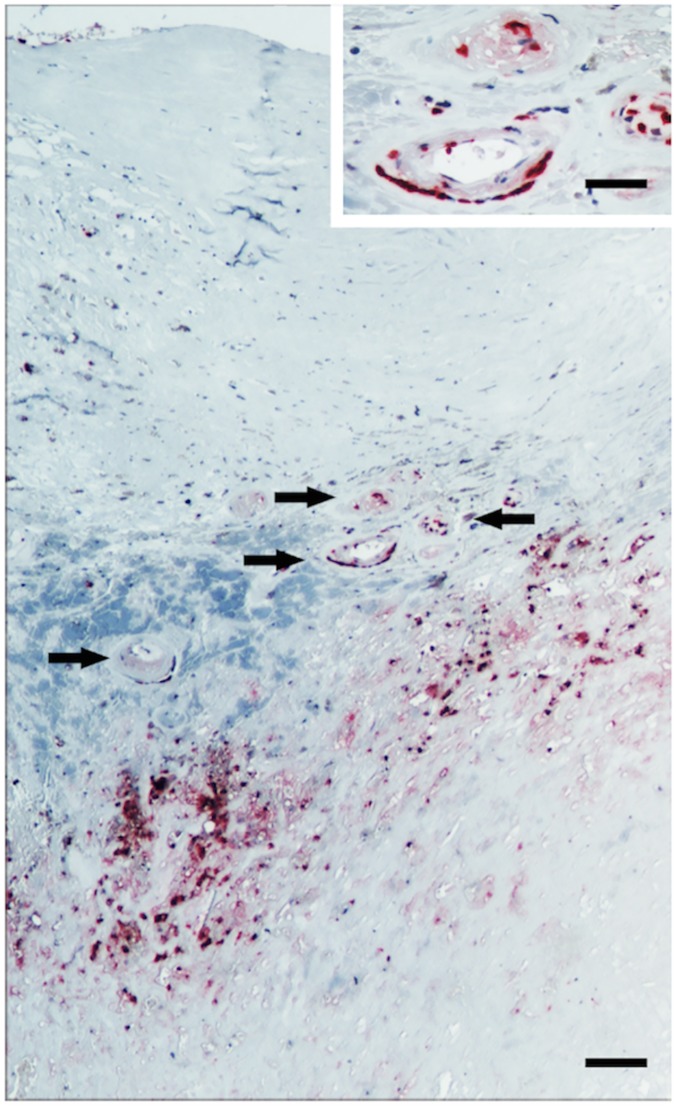
Immunohistochemical staining of CD66b+ cells in a plaque specimen. Cells are clearly localized in areas adjacent to the plaque and usually close to the neovessels (arrows). Bar: 100 μm (insert: 40 μm).

Immunohistochemical analysis was performed on samples from 8 soft and 5 mix plaques (Table 6). Both soft and mix plaque specimens showed massive presence of infiltrating immune cells, including CD3+ T lymphocytes, CD163+ monocytes, and CD68+ macrophages (data not shown). Immunostaining for CD66b showed that CD66b+ cells were preferentially localized only in areas with massive plaque formation. No CD66b+ cells were found in areas adjacent to the plaque and corresponding to common carotid artery and to internal carotid artery. Most CD66b+ cells were localized in the areas of maximum stenosis, usually close to the neovessels (Fig 1). No significant differences occurred in the density of CD66b+ cells between soft and mix plaques measured as cells/field (Table 6).

**Table 6:** Immunohistochemistry of plaque specimens and density of CD66b+ cells.

	Total	Soft	Mix
**CD66b+ cells/field**			
0	2	1	1
1–9	2	2	0
10–50	4	1	3
>50	5	3	2

Values indicated in the table represent absolute values.

### PMN recovery from plaques

After mechanical disaggregation of the whole specimens, cell number in tissue homogenate was counted before and after the immunomagnetic cell sorting. PMN recovering from homogenates was 2.34±2.56x10^7^ and, after sorting, highly purified PMN were 1.23±1.56x10^6^. No differences in cell number was observed between soft and mixed plaque in both homogenate and purified PMN (P always >0.05).

### mRNA expression of IL-8, VEGF and elastase by cPMN and pPMN

#### IL-8

**Fig 2:**
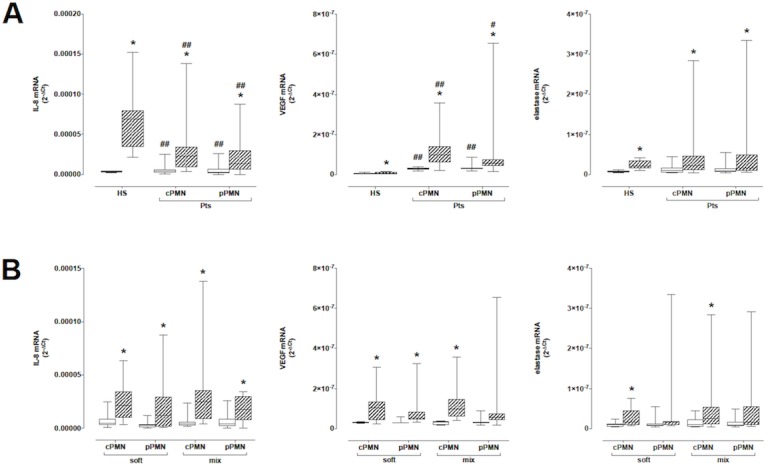
IL-8 (left panel), VEGF (middle) and elastase (right) mRNA expression in PMN at rest (empty columns) and after stimulation with fMLP (hatched columns). Panel A: Comparison between HS and Pts. Panel B: Comparison between Pts with soft and mix plaques. * = P<0.05 vs resting; # = P<0.05 and ## = P<0.01 vs HS.

In resting PMN of HS IL-8 mRNA levels were 3.58 (3.02–4.24) x 10^–6^ and underwent an about 20-fold increase after stimulation with fMLP. Both cPMN and pPMN from Pts expressed significantly higher levels of IL-8 mRNA which however increased less after fMLP, thus resulting in stimulated levels which were significantly lower than those in PMN of HS (Fig 2, panel A, left). No significant differences occurred in IL-8 mRNA levels in either cPMN or pPMN between Pts with soft and mix plaques (Fig 2, panel B, left).

#### VEGF

Levels of VEGF mRNA in PMN of HS were 3.33 (2.47–7.39) x 10^–9^ and showed a slight although statistically significant nearly 2-fold increase after fMLP, up to 7.49 (2.47–7.39) x 10^–9^. By comparison, VEGF mRNA levels were significantly higher in resting cPMN and pPMN and after fMLP they strongly increased, resulting in mRNA levels about 11.8–13.9 fold higher than in stimulated PMN of HS (Fig 2, panel A, middle). VEGF mRNA levels were similar in both cPMN and pPMN from Pts with soft and mix plaques and increased to about the same extent after fMLP (Fig 2, panel B, middle).

#### Elastase

Levels of elastase mRNA were not significantly different in PMN from HS and in cPMN and pPMN from Pts, and significantly increased to about the same extent after fMLP (Fig 2, panel A, right). There were no significant differences in elastase mRNA levels in cPMN and pPMN between Pts with soft and mix plaques (Fig 2, panel B, right).

IL-8, VEGF and elastase mRNA levels did not differ in either cPMN or pPMN according to the absence or presence of cerebrovascular symptoms (not shown).

### Protein production of IL-8, VEGF and elastase

#### IL-8

**Fig 3:**
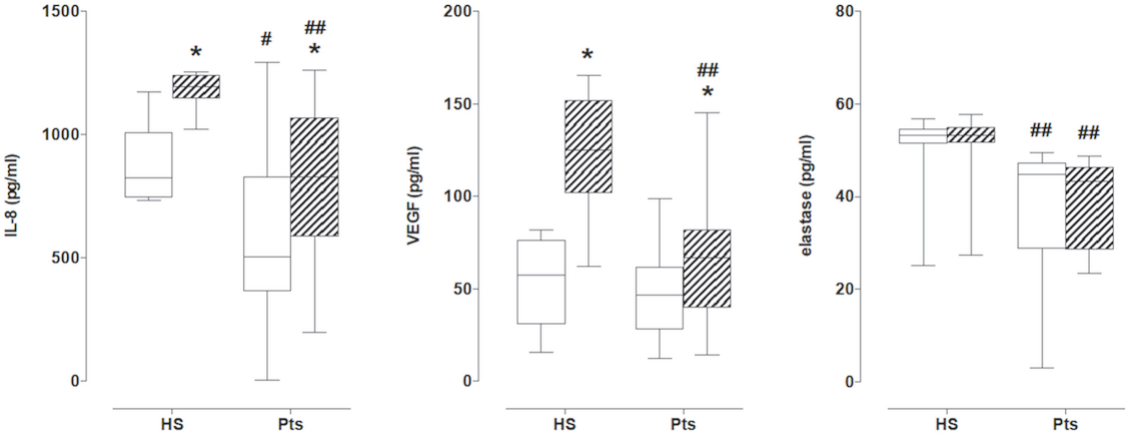
IL-8 (left), VEGF (middle) and elastase (right) production in cPMN of patients (Pts) and healthy subjects (HS) at rest (empty columns) and after stimulation with fMLP (hatched columns). * = P<0.05 vs resting; # = P<0.05 and ## = P<0.01 vs HS.

Both resting and stimulated IL-8 levels were higher in cPMN of HS with respect to levels in cPMN of Pts (Fig 3, left panel). Both resting and stimulated IL-8 production were higher in cells of Pts with soft plaques with respect to values measured in cells of Pts with mixed plaques (Table 7). Resting IL-8 production was also lower in cells from Pts with cerebrovascular symptoms (258.9 [73.0–645.1] vs 534.6 [461.8–868.1], P = 0.03).

**Table 7:** IL-8, VEGF and elastase protein production in cPMN of Pts with soft and mixed plaques.

Soft plaques			mixed plaques	
	Resting	fMLP-induced	Resting	fMLP-induced
IL-8 (pg/ml)	397.5 (161.8–662.6)	832.2 (410.2–1005.0)*	525.5 (387.0–918.4)^#^	794.2 (643.5–1160.0)**
VEGF (pg/ml)	38.3 (28.5–57.0)	65.0 (38.8–83.1)**	53.0 (34.7–64.0)	67.4 (44.0–95.1)
elastase (ng/ml)	44.7 (28.4–46.6)	41.8 (28.6–45.0)	46.9 (44.0–48.5)	46.1 (43.3–48.1)

Data are expressed as median (25^th^-75^th^ percentile).

* = P<0.05.

** = P<0.005 vs respective control

# = P< 0.05 resting mixed vs resting soft.

#### VEGF

No differences were observed in resting production between HS and Pts, while stimulated production was higher in HS with respect to Pts (Fig 3, middle panel). No differences occurred in resting conditions between cells of Pts with soft and mixed plaques, while stimulation with fMLP did not increased VEGF production in Pts with mixed plaques (Table 7). There were no differences between cells from Pts with and without cerebrovascular symptoms (not shown).

#### Elastase

No differences between resting and stimulated conditions were found between cells of HS and of Pts, although elastase production was always higher in PMN of HS (Fig 3, right panel). Elastase production was similar in cells of Pts with soft and mixed plaques (Table 7). No differences were found between cells from Pts with and without cerebrovascular symptoms (not shown).

### Correlation between PMN production of IL-8, VEGF and elastase and Pts clinical profile

No correlations were found either in total Pts or in Pts with soft or mix plaques with clinical characteristics (Table 2), drug treatments (Table 3), or data at enrollment (Table 4).

## Discussion

Although the role of chronic inflammation of the vascular wall in ATH is now well established [35], only recently evidence has been provided regarding the possible causative role of PMN in the development and progression of ATH. In ApoE(-/-) mice, PMN are a major cellular component of atherosclerotic lesions, where they migrate in response to a high-fat diet during early stages of ATH [36] and where they represent a majority of leukocytes interacting with endothelium [37]. In this animal model, depletion of PMN strongly reduces the area of ATH lesions, indicating a crucial role in plaque formation [36]. In humans, PMN have been identified in coronary plaques, where they are associated with acute coronary events [14], and in carotid plaques, where their numbers are strongly associated with the histopathologic features of rupture-prone plaques, suggesting a role for PMN in plaque destabilization [38].

By use of different approaches, we have now provided an extensive functional characterization of PMN infiltrating carotid plaques in comparison to circulating PMN, regarding their ability to produce key mediators of the atherosclerotic process, namely IL-8, VEGF and elastase. In particular, cells were investigated both at rest and after stimulation, thus providing detailed information about their full potential responsiveness after activation. In addition, by use of immunohistochemistry we confirmed the presence of CD66b+ cells in CEA specimens and their preferential localization in close proximity of microvessels.

Our results indicate that both circulating and intraplaque PMN from subjects with carotid ATH are active producers of IL-8, VEGF and elastase and in particular that intraplaque PMN likely represent important local sources of such mediators. Moreover, evidence is provided that these PMN, in comparison to cells from healthy subjects, have, at least at mRNA levels, decreased ability to produce IL-8 but increased ability to produce VEGF, while the production of elastase is not significantly different between patients and healthy subjects. Decreased IL-8 mRNA and increased VEGF mRNA occur in both intraplaque and circulating PMN, at rest as well as after stimulation, suggesting that such functional changes are systemic and not limited to cells infiltrating the vascular wall.

Interestingly, measuring protein production in circulating cells, we showed that cPMN of Pts produce less IL-8, elastase and VEGF with respect to HS. This unexpected evidence deserve additional investigations, because we cannot explain this apparent discrepancy; a first explanation can come from the fact that these Pts are all in therapy from different years with a lot of drug acting at different levels and known to be affecting immune response, but to our knowledge no data are present in literature about a different modulation of cell product at mRNA and protein levels by these drugs. Our previous data on Pts with peripheral artery disease (PAD) shows that IL-8 production in PMN of Pts was lower with respect to HS, and also in this case, Pts were all in polytherapy, but no data were obtained about the mRNA expression [21]. We cannot exclude that drugs taken from Pts can differently modulate cellular VEGF or elastase and IL-8 production resulting in this apparent opposite data at mRNA and protein levels.

Decreased ability of PMN to produce IL-8 is in agreement with previous findings of our group, showing decreased production of IL-8 in circulating PMN from patients with peripheral arterial disease (PAD) undergoing femoral endarterectomy [21]. In that study, patients with PAD were monitored after surgery, and PMN production of IL-8 did not recover over a period of at least 6 months. Ionita et al. [38] by use of immunohistochemistry reported that in human carotid plaques high PMN numbers were associated with high IL-8 in the vascular wall. In the light of our present results, it may be suggested that increased IL-8 in atherosclerotic lesions is the result of PMN accumulation in the plaque rather than of the enhanced ability of individual cells to produce this chemokine. Interestingly, IL-8 production by circulating PMN is increased in healthy subjects at high cardiovascular risk [7], in the context of an apparently generalized increased activity of these cells which includes increased oxidative metabolism [7] and increased angiotensin II type 1 receptor (AT_1_R) expression [8]. Antidyslipemic treatment with statins is indeed able to reverse at least in part enhanced PMN activity in high-risk subjects (a few weeks of simvastatin resulting in decreased IL-8 production and AT_1_R expression, but not in reduction of the oxidative metabolism) [7,8], thus it could be proposed that also in subjects with established ATH statin treatment provides a major contribution to the impairment of IL-8 production by PMN, according to the present data showing that IL-8 protein production was lower in Pts with respect to HS. In our study however IL-8 levels were not significantly different in PMN from patients who received statins in comparison to those inc ells from patients who were not on statins, and actually, at least in stimulated circulating PMN, IL-8 mRNA and not protein levels were even higher in subjects receiving statins. Additional investigations are therefore required to identify factors involved in the impaired ability of PMN to produce IL-8 in established ATH as well as its implications for plaque development and progression. In any case the present results confirm and extend previous report concerning the ability of intraplaque PMN to produce IL-8 [38], thus indicating that local production in atherosclerotic lesions is brought about not only by endothelial cells but also by PMN infiltrating the vascular wall.

Increased ability to produce VEGF by PMN in ATH to our knowledge was never reported before and deserves consideration. VEGF is a major angiogenic factor, regulating vascular growth, function, and homeostasis, permeability and vasodilatation. In ATH, normal angiogenesis restores vessel wall normoxia and contributes to the resolution of inflammation, leading to disease regression. On the contrary, pathological angiogenesis enhances ATH progression, increasing macrophage infiltration and vessel wall thickness, perpetuating hypoxia and necrosis. In particular, neoangiogenesis of plaque lesions favors their progression toward rupture [26,39]. It has been recently suggested that smooth muscle cells located underneath early atheromatous lesions, stimulated by the accumulation of lipids in the arterial wall, may become major sources of VEGF thus acquiring a pro-angiogenic phenotype and eventually promoting intraplaque angiogenesis [39]. Our findings add to this picture, increasing its complexity by showing that also infiltrating PMN contribute to the local production of VEGF. In this regard, it must not be neglected that even IL-8 is a potent angiogenic factor and that circumstantial evidence suggests its contribution in plaque formation via its angiogenic properties [40]. As a whole, our results point to PMN infiltrating atherosclerotic plaques as an important source of VEGF and IL-8, which in turn provide a major contribution to plaque development and progression through both their proangiogenic effects and their chemotactic activity towards inflammatory cells such as monocytes/macrophages and PMN themselves.

The presence within atherosclerotic plaques of the proteolytic enzyme elastase is well established. Elastase contributes to matrix degradation and weakening of the vessel wall ultimately leading to aneurysm formation and plaque rupture. So far however accumulation of elastase in the vessel wall was mainly attributed to peripheral blood-derived monocytes and possibly by endothelial cells [18]. Only in vitro evidence was so far available regarding the possibility that IL-8 dependent recruitment of PMN to the vascular wall finally results in the local production of potent matrix-degrading enzymes (such as elastase and matrixmetalloproteinase-8 (MMP-8)) and in the induction of endothelial cell apoptosis [41]. The present results represent therefore the first direct evidence that elastase can be directly produced by intraplaque PMN. Ionita et al.[38] showed that high levels of MMP-8 are directly correlated with high PMN numbers in carotid plaques, thus strengthening the hypothesis that intraplaque PMN may be key local producers of matrix-degrading enzymes with critical implications for plaque destabilization and subsequent cardiovascular events.

An additional aim of our study was to evaluate any possible correlations between PMN and the characteristics of the plaques. In this regard, we observed no major differences in the functional profile of either circulating or intraplaque PMN between patients with soft and mix plaques, however it was of interest that patients with soft plaques had significantly higher white blood cells with a more than 25% increase in the absolute count of circulating PMN. The density of PMN in plaque specimens on the contrary was not significantly different between soft and mixed plaques, however the immunohistochemical analysis was essentially qualitative and therefore intrinsically limited and not suitable for any quantitative assessment. Ionita et al. [38] reported that in carotid plaque specimens high microvessel density within plaques was correlated with high PMN numbers, an observation which likely points to a relationship between PMN infiltration and plaque architecture. Also in view of the similar functional profile of circulating and intraplaque PMN, we propose at least as a working hypothesis that a higher number of circulating PMN provides an increased pool of cells potentially available to migrate in the vascular wall. It remains to be established whether higher microvessel density is a preexisting factor resulting in enhanced PMN migration in the plaque or it is also possibly a consequence of the increased number of intraplaque PMN which locally produce and release direct and indirect proangiogenic factors like IL-8, VEGF and elastase.

In conclusion, the present study provides a functional characterization of PMN in patients with carotid atherosclerotic plaques, showing that both circulating as well as intraplaque PMN are able to actively produce crucial factors affecting plaque development and progression, such as IL-8, VEGF and elastase. Circulating PMN count has been recently shown to be an independent predictor for all-cause and cardiovascular mortality in patients with carotid ATH [42], and the present results contribute to provide mechanistic explanations to such observations. Clarifying the role of PMN in ATH and in particular their specific contribution to plaque development and eventually to its destabilization will provide further insights into plaque biology and will hopefully indicate novel pharmacotherapeutic strategies to interfere with plaque progression and related complications.

## Supporting Information

S1 Data Information about raw data. (XLSX)
