# Correction: Production of IL-8, VEGF and Elastase by Circulating and Intraplaque Neutrophils in Patients with Carotid Atherosclerosis

**DOI:** 10.1371/journal.pone.0181389

**Published:** 2017-07-10

**Authors:** Franca Marino, Matteo Tozzi, Laura Schembri, Stefania Ferraro, Antonino Tarallo, Angela Scanzano, Massimiliano Legnaro, Patrizio Castelli, Marco Cosentino

## Notice of Republication

This article was republished on May 22, 2017, as the original S1 Dataset was published in error. The correct Data Availability statement is: Due to ethical restrictions pertaining to participant privacy, data from this study are available upon request to the corresponding author at franca.marino@uninsubria.it. Please download this article again to view the correct version.
